# Influence of tumour location on the survival outcomes of upper tract urothelial carcinoma treated with radical nephroureterectomy

**DOI:** 10.1007/s00345-024-05432-0

**Published:** 2025-05-03

**Authors:** Kang Liu, David Ka-Wai Leung, Chris Ho-Ming Wong, Ivan Ching-Ho Ko, Rahim Horuz, Paolo Gontero, Pilar Laguna, Jean de la Rosette, Jeremy Yuen-Chun Teoh

**Affiliations:** 1 Department of Surgery, S.H. Ho Urology Centre, The Chinese University of Hong Kong, Hong Kong, China; 2 International School of Medicine, Istanbul Medipol University, Istanbul, Turkey; 3 Department of Urology, Istanbul Medipol Mega University Hospital, Istanbul, Turkey; 4 Department of Urology, University of Turin, Turin, Italy; 5 Department of Urology, Medical University of Vienna, Vienna, Austria

**Keywords:** Upper Tract Urothelial Carcinoma, Location, Radical Nephroureterectomy, Survival

## Abstract

**Objective:**

To evaluate the impact of tumour location on the survival outcomes of patients with upper tract urothelial carcinoma (UTUC) after radical nephroureterectomy (RNU).

**Method:**

Patients with ureteral urothelial carcinoma (UUC) or renal pelvic urothelial carcinoma (RPUC) of the Clinical Research Office of the Endourology Society (CROES)-UTUC registry were analyzed. Study outcomes included overall survival (OS), cancer-specific survival (CSS), intravesical recurrence-free survival (IRFS), and progression-free survival (PFS), which were compared using Kaplan–Meier method with log-rank test. Propensity score matching (PSM) was performed to balance the differences in tumour features between the two groups.

**Result:**

The UUC and RPUC groups consisted of 309 (41.9%) and 429 (58.1%) patients, respectively. RPUC group had larger tumour size (77.9% ≥ 2 cm vs 67.0% in UUC, p < 0.01), and more T3/T4 tumours (36.4% vs. 22. 0%, p < 0.01). The UUC group exhibited worse PFS compared to the RPUC group ( p = 0.029 for the initial analysis and p = 0.013 after PSM). However, there were no significant differences in OS (p = 0.088 before PSM and p = 0.255 after PSM), CSS (p = 0.106 before PSM and p = 0.101 after PSM), or IRFS (p = 0.112 before PSM and p = 0.28 after PSM) between the two groups.

**Conclusion:**

Patients with ureteral urothelial carcinoma exhibited worse PFS compared to those with renal pelvic urothelial carcinoma. However, no significant differences were observed in OS, CSS, or IRFS between the two tumour locations. UTUC patients should be counselled about their individualized prognosis accordingly.

**Registration:**

NCT02281188.

**Supplementary Information:**

The online version contains supplementary material available at 10.1007/s00345-024-05432-0.

## Introduction

Upper tract urothelial carcinoma (UTUC) refers to urothelial tumour that originates in the renal pelvis, the ureter, or both. Although UTUC is a rare tumour, accounting for only five to ten percent of all urothelial carcinomas [1], it is relatively more aggressive than its bladder counterpart, as two-thirds of UTUC cases present with invasive disease [2]. The standard treatment for high-risk UTUC patients is radical nephroureterectomy (RNU) with bladder cuff excision, while their age, comorbidities, and personal preferences also need to be taken into consideration [3]. However, the long-term survival remains poor, with a 5-year cancer-specific survival (CSS) rates ranging from 44 to 86%, depending on the tumour stage [2]. The known prognostic factors include tumour stage and grade, tumour multifocality, tumour size, hydronephrosis, local invasion, and variant histology [2]. However, there is ongoing controversy about how tumor location may affect the oncological outcomes for UTUC, with a lack of consistent results across studies.

As such, we conducted this retrospective analysis of a multi-institutional global registry, to further evaluate the survival outcomes of ureteral urothelial carcinoma (UUC) versus renal pelvic urothelial carcinoma (RPUC), and to provide additional updated evidence to guide clinical practice.

## Method

### Population

All the eligible patients from the Clinical Research Office of the Endourology Society (CROES)-UTUC registry were included in the current study. The exclusion criteria encompass the following: 1. Patients who do not undergo RNU; 2. Patients with unavailable survival outcomes; 3. Patients with an unspecified tumour location or those presenting with dual tumour sites. The CROES-UTUC registry was an international cohort which prospectively collected data of UTUC patients from 101 centres of 29 countries [4]. It provides the latest data about the risk factors, clinical management [5, 6], and outcomes [7] of UTUC by recording comprehensive real-world data. Data from all the participating centers were collected using an online Data Management System. The Data Management System was a web-based system located and maintained at the CROES Office. The registry follows the recommendations of the Agency for Healthcare Research and Quality for the design and use of patient registries for scientific, clinical, and health policy purposes. The study was registered with clinicaltrials.gov (NCT02281188).

### Patient inclusion

We included all the histologically confirmed UTUC patients treated with RNU from the CROES registry, given that the tumor location was known, and follow-up data were available. Cases with unknown or mixed (both pelvic and ureteral) tumor locations were excluded.

### Outcomes

Survival outcomes including overall survival (OS), cancer-specific survival (CSS), intravesical recurrence-free survival (IRFS), and progression-free survival (PFS), were compared between patients with renal pelvis and ureteral urothelial carcinoma.

### Statistical analysis

The categorical variables were depicted in numbers and percentages, whereas the continuous variables were expressed in medians with interquartile range [IQR]. The Chi-square test for categorical variables and the Mann–Whitney test for continuous parameters were employed to compare the two groups. Survival outcomes were analyzed using Kaplan–Meier plots and the log-rank test. Propensity scores matching (PSM) was performed with a randomization of case order, and priority to nearest matches. The matching tolerance was set at 0.1, meaning that an adjusted mean difference (AMD) of less than 10% is generally considered indicative of good matching quality. The parameters used in the propensity scores matching consist of tumour stage, tumour grade, and tumour size, tumour side, and tumour multifocal rates. Some data was missing, and we assumed that the missing pattern was completely at random and would not affect our further analysis. All the statistical analyses were performed using SPSS and “jskm” package of R software (4.2.0; R Foundation for Statistical Computing, Vienna, Austria).

## Result

### Patient demographics

There were 2380 patients of the CROES-UTUC registry in total. We excluded 851 patients without RNU treatment, 583 patients without survival outcomes, 121 patients without information of tumour location, and 87 patients with both RPUC and UUC. Finally, 738 patients were enrolled in the analysis, consisting of 309 (41.9%) UUC and 429 (58.1%) RPUC (Supplementary Fig. 1).

There were not statistically significant differences between UUC and RPUC in terms of gender (p = 0.93), age (p = 0.93), smoking status (p = 0.55), race (p = 0.87), family history (p = 0.90), contralateral UTUC (p = 0.73), solitary kidney rate (p > 0.99), ASA class (p = 0.29), and adjuvant chemotherapy (p = 0.83). However, UUC group had high proportion of concomitant bladder cancer (19.1% vs 8.9% in RPUC, p < 0.01) and positive margin rates (13.6% vs 3.0% in RPUC, p < 0.01). As for tumour factors, UUC and RPUC had similar disease side distributions (p = 0.38), tumour multifocal rates (p = 0.87), and tumour grade (p = 0.84). Notably, the RPUC group had larger tumour size (77.9% $\ge$ 2 cm vs 67% in UUC, p < 0.01) and higher tumour stage (36.4%$\ge$ T3 vs 22.0% in UUC, p < 0.01) (Table 1).

**Table 1:** Baseline characteristics of ureteral urothelial carcinoma (UUC) and renal pelvic urothelial carcinoma (RPUC)

Characteristic	UUC	RPUC	p value
	n = 309	n = 429	
Gender, n (%)			
Male	224 (72.5)	309 (72.0)	0.93
Female	85 (27.5)	119 (27.8)	
Missing	0	1 (0.2)	
Age (yr), median [IQR]	71.0 [63.0–77.0]	71.0 [63.5–77.5]	0.93
Smoking, n (%)			
No	88 (28.5)	139 (32.4)	0.55
Ex-smoker	111 (35.9)	143 (33.3)	
Current smoker	77 (24.9)	108 (25.2)	
Missing	33 (10.7)	39 (9.1)	
Race, n (%)			
White	223 (72.3)	315 (73.4)	0.87
Asian	61 (19.7)	78 (18.2)	
Other	19 (6.1)	26 (6.1)	
Missing	6 (1.9)	10 (2.3)	
Family history, n (%)			
No	230 (74.4)	324 (75.6)	0.90
Yes	17 (5.5)	25 (5.8)	
Missing	62 (20.1)	80 (18.6)	
Contralateral UTUC, n (%)			
No	304 (98.4)	425 (99.1)	0.73
Yes	4 (1.3)	4 (0.9)	
Missing	1 (0.3)	0	
Solitary kidney, n (%)			
No	308 (99.7)	428 (99.8)	> 0.99
Yes	0 (0.0)	1 (0.2)	
Missing	1 (0.3)	0	
Concomitant bladder cancer, n (%)			
No	187 (60.5)	284 (66.2)	< 0.01
Yes	59 (19.1)	38 (8.9)	
Missing	63 (20.4)	107 (24.9)	
ASA score, n (%)			
0	33 (10.7)	61 (14.2)	0.29
1	151 (48.9)	216 (50.4)	
2	110 (35.5)	130 (30.3)	
3	7 (2.3)	13 (3.0)	
Missing	8 (2.6)	9 (2.1)	
Positive margin, n (%)			
No	222 (71.8)	369 (86.0)	< 0.01
Yes	42 (13.6)	13 (3.0)	
Missing	45 (14.6)	47 (11.0)	
Adjuvant chemotherapy, n (%)			
No	281 (90.9)	389 (90.7)	0.83
Yes	24 (7.8)	30 (7.0)	
Missing	4 (1.3)	10 (2.3)	
Side, n (%)			
Left	167 (54.0)	218 (50.8)	0.38
Right	139 (45.0)	206 (48.0)	
Both	3 (1.0)	5 (1.2)	
Missing	0	0	
Tumour Size, n (%)			
< 2 cm	88 (28.5)	75 (17.5)	< 0.01
≥ 2 cm	207 (67.0)	334 (77.9)	
Missing	14 (4.5)	20 (4.6)	
Tumour Focality, n (%)			
Unifocal	277 (89.6)	383 (89.3)	0.87
Multifocal	32 (10.4)	46 (10.7)	
Missing	0	0	
Tumour Grade, n (%)			
G1	44 (14.2)	55 (12.8)	0.84
G2	69 (22.3)	100 (23.3)	
G3	160 (51.8)	225 (52.4)	
Missing	36 (11.7)	49 (11.4)	
Tumour Stage, n (%)			
Ta	64 (20.7)	99 (23.1)	< 0.01
Tis	7 (2.3)	4 (0.9)	
T1	68 (22.0)	83 (19.3)	
T2	77 (24.9)	58 (13.5)	
T3	63 (20.4)	138 (32.2)	
T4	5 (1.6)	18 (4.2)	
Missing	25 (8.1)	29 (6.8)	

There were 155/738 (21.0%) patients were omitted in PSM due to the missing data of tumour stage, tumour grade, and tumour size, tumour side, tumour multifocal rates, and positive margin rates. The concomitant bladder cancer rate was not included in the PSM due to the missing data rate being too high.

### Survival outcomes

**Fig. 1:**
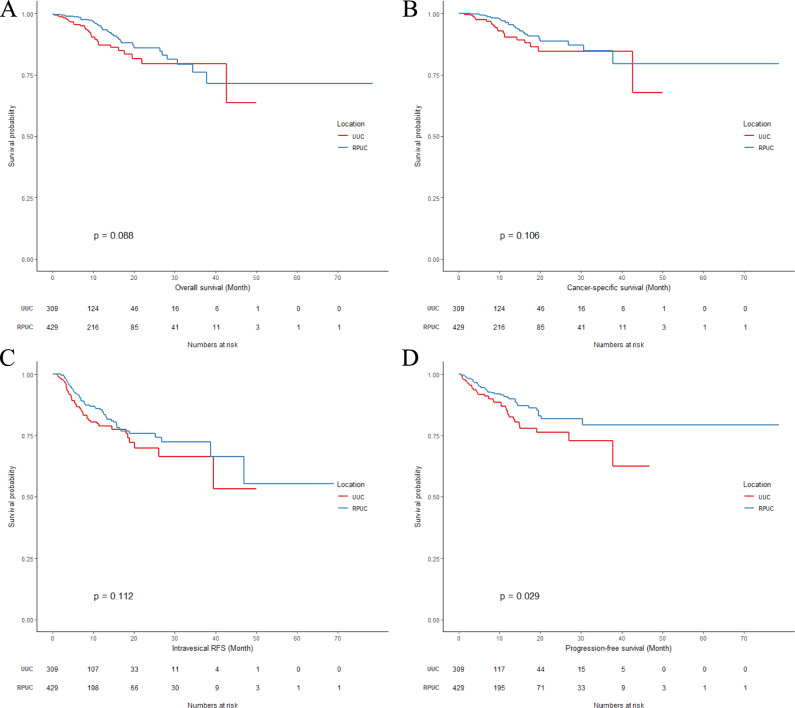
Survival outcomes. **A** OS, **B** CSS, **C** IRFS, **D** PFS

Before PSM, the Kaplan–Meier plots revealed that UUC had worse PFS (p = 0.029) than RPUC, whilst OS (p = 0.088), CSS (p = 0.106), and IRFS (p = 0.112) were similar between the two groups (Fig. 1).

**Fig. 2:**
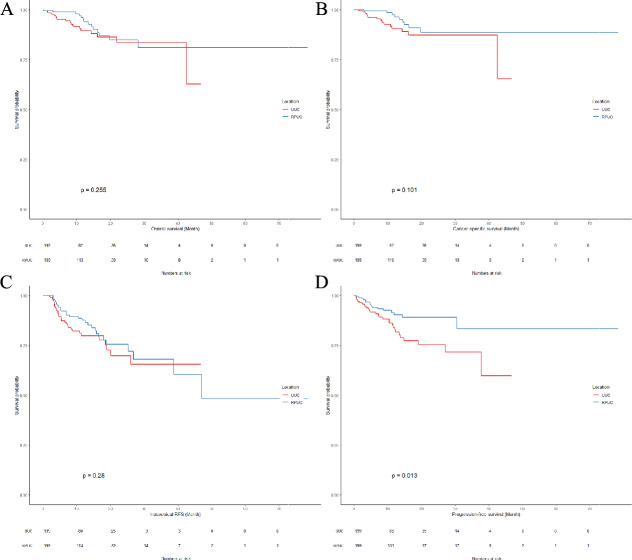
Survival outcomes after PSM. **A** OS, **B** CSS, **C** IRFS, **D** PFS

After PSM, the two groups had balanced tumour features (Table 2, Supplementary Fig. 2) as well as other clinical characteristics except concomitant bladder cancer rates (Supplementary Table 1), and the significant difference in PFS continued to favor the renal pelvis subgroup (p = 0.013). There were no significant differences in OS (p = 0.255), CSS (p = 0.101), and IRFS (p = 0.28) between UUC and RPUC (Fig. 2).

**Table 2:** The tumour features after PSM

Characteristic	UUC	RPUC	AMD
	n = 199	n = 199	
Positive margin, n (%)			
No	191 (96.0)	189 (95.0)	0.01
Yes	8 (4.0)	10 (5.0)	0.01
Tumour Side, n (%)			
Left	104 (52.3)	104 (52.3)	< 0.01
Right	93 (46.7)	94 (47.2)	< 0.01
Both	2 (1.0)	1 (0.5)	< 0.01
Tumour Size, n (%)			
< 2 cm	48 (24.1)	52 (26.1)	0.02
≥ 2 cm	151 (75.9)	147 (73.9)	0.02
Tumour Focality, n (%)			
Unifocal	183 (92.0)	186 (93.5)	0.02
Multifocal	16 (8.0)	13 (6.5)	0.02
Tumour Grade, n (%)			
G1	29 (14.6)	26 (13.1)	0.02
G2	51 (25.6)	48 (24.1)	0.02
G3	119 (59.8)	125 (62.8)	0.03
Tumour Stage, n (%)			
Ta	36 (18.2)	32 (16.2)	0.02
Tis	2 (1.0)	2 (1.0)	< 0.01
T1	53 (26.6)	59 (29.6)	0.03
T2	58 (29.1)	49 (24.6)	0.04
T3	47 (23.6)	52 (26.1)	0.03
T4	3 (1.5)	5 (2.5)	0.01

*AMD* Adjusted mean difference

## Discussion

The current literature suggests that large tumor size [8], multifocality [9, 10], hydroureteronephrosis [11], high tumor grade and stage [12] are all factors predicting adverse oncological outcomes. However, previous studies about tumor location as a prognostic factor have reported conflicting results. Raman et al. reported that there was no difference in recurrence or cancer-specific mortality rates between UUC and RPUC [13]. Contrarily, a retrospective cohort study using 1995–2010 data suggested that the UUC group had worse cancer-specific survival and metastasis rates but similar OS, compared to RPUC even when adjusted for stage [9].

A recent study that included 476 patients with pT2N0M0 localized UTUC found that ureteral tumors exhibited a worse 5-year RFS, CSS and OS than pelvic tumors [14]. Another study that analyzed 302 pT3N0M0 UTUC patients treated with RNU showed that ureteral location was associated with twice the risk of local recurrence compared to that of pelvic location, upon multivariate Cox regression analyses [15]. Our PSM analyses produced similar findings of these newer studies and affirmed the negative impact of ureteral location on the PFS of UTUC. However, the better PFS of RPUC did not correlate with an improvement in OS, which possibly reflecting the increased risk of death from other comorbidities among elderly UTUC patients.

In order to explore the mechanisms that underlie the aggressiveness of UTUC, especially ureteral ones, its anatomy and pathophysiology must first be addressed. The modes of spread of UTUC constitutes of (1) direct invasion into the renal parenchyma or surrounding structures (2) lymphatic and hematogenous invasion, and (3) bladder urothelium reimplantation resulted from downstream seeding.

Gaining more knowledge in the potential implications of tumour location on management outcomes may assist in treatment planning and patient counselling. One of the key parameters is the implication of tumour location on margin status. In our analysis, we noticed a higher margin positive rates for UUC compared to RPUC, even after tumour stages being balanced in PSM. Multiple studies [16, 17] have demonstrated that margin is a key predictor of oncological outcomes, whether assessed singularly or as a constituent of a “fecta” [18] based criteria. Additionally, a lower threshold may be considered for ureteric location of tumour in terms of initiating neoadjuvant chemotherapy. RCT has demonstrated a partial pathological response of NAC of 63% and complete response of 19% [19]. It would be interesting to further accumulate real-world data and observe if there is an interplay between tumour location, NAC and oncological outcomes.

Out of the three proposed mechanisms, the direct invasion likely weights most significant in terms of disease progression and the subsequent cancer-related mortality. The epithelial lining of the renal calyx, renal pelvis, ureters, bladder and urethra are all made of urothelium [20]. Histologically, the renal calyces and ureters contain two layers of smooth muscle, surrounded by the serosa made up of blood vessels and lymphatics. Compared to the bladder, the upper tract muscularis propria is characteristically thinner. It would be reasonable to expect that UUC would be associated with higher risks of more advanced stages (T3 + disease) and extra-ureteral extension [21, 22].

Whether this difference of susceptibility in local invasiveness materialises to differences in long term oncological outcomes remain inconclusive. Perez-Montiel et al. reported from their clinicopathological analyses of 109 cases of high-grade renal pelvis UTUC that, among the pT3 cases, the patients who died of the cancer had tumours that extensively infiltrated the renal parenchyma; whereas those who survived the disease only had focally infiltrative disease [23]. This may constitute the postulation of the protective effect of renal parenchyma of RPUC against local disease spread. This also echoed with our current analysis, which demonstrated inferior PFS (hence higher likelihood of progression) in UUC in a propensity-matched cohort. Whether it conveys to OS difference remains unanswered and evidence were conflicting. A population-based analysis reported less adoption of RNU and inferior OS for UUC compared to RPUC [24]. Meanwhile, a number of retrospective cohorts have failed to demonstrate a relationship between tumour location and survival outcomes [25, 26]. Further studies about the anatomical, pathological, lymphatic or hematogenous spread and possibly genetic aspects of UTUC are certainly needed to fully explain the difference in clinical behaviour between RPUC and UUC.

### Limitations

This study has several limitations. First, although data was prospectively and consecutively collected, definitive objectives were not formulated at registry inception, and thus a selection by inclusion of only patients with data available to fulfill the present comparative purposes. Nonetheless, owing to its rarity, prospective multicenter registries may well be the most feasible data in this field. Second, there were some missing data on patients’ tumour features that reduced the sample size after PSM. The concomitant bladder cancer rate was not included in the PSM due to the missing data rate being too high. As cases were included based on the availability of complete data rather than random sampling. This can limit generalizability since the data may not fully represent all UTUC patients treated with RNU. Although PSM adjusting, there was heterogeneity in the clinical management among the different centers participating in the registry and even in the present cohort. Besides, follow-up protocols likely varied across centers, with differences in surveillance intensity or treatment decisions possibly affecting outcomes like PFS and IRFS. Despite these limitations, our data provides contemporary real-world information originated through multiple practices in different countries, and may contribute to better information on stratification and management of UTUC.

## Conclusion

Patients with ureteral urothelial carcinoma have a worse PFS compared to those with renal pelvic urothelial carcinoma. However, no significant differences were observed in OS, CSS, or IRFS between the two tumour location groups. More individualized prognosis depending on the tumour location should be included in patient counselling. Further studies are required to elucidate the underlying mechanism to explain the noted difference in PFS.

## Supplementary Information

Below is the link to the electronic supplementary material.

Supplementary file1 (DOCX 189 KB)

Supplementary file2 (DOCX 18 KB)

## Data Availability

The datasets used and/or analyzed during the current study are available from the corresponding author on reasonable request.
