# Supplementary material for: Influence of tumour location on the survival outcomes of upper tract urothelial carcinoma treated with radical nephroureterectomy
Source: World J Urol. 2025 May 3;43(1):261. doi: 10.1007/s00345-024-05432-0 (PMC12049378; doi:10.1007/s00345-024-05432-0)

**Supplementary Figure 1. Flow chart.**


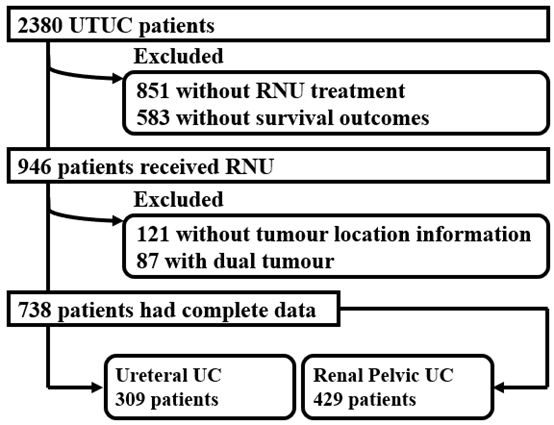


**Supplementary Figure 2. Selected cases of PSM. A) Jitter chart, B) Bar chart.**


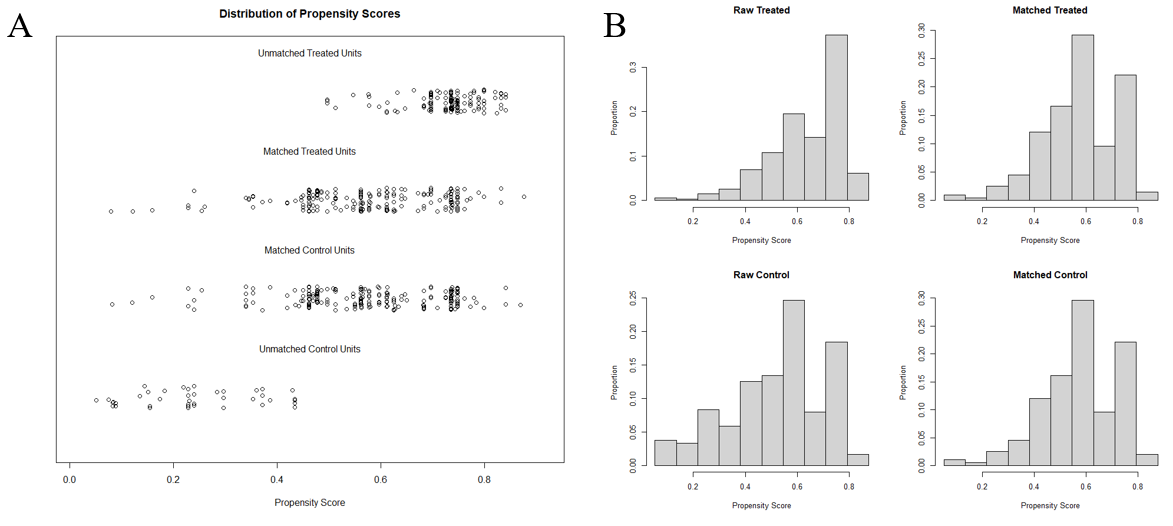

Supplement: Supplementary file 1 — Supplementary file1 (DOCX 189 KB) [file 345_2024_5432_MOESM1_ESM.docx]
