# Supplementary material for: Influence of tumour location on the survival outcomes of upper tract urothelial carcinoma treated with radical nephroureterectomy
Source: World J Urol. 2025 May 3;43(1):261. doi: 10.1007/s00345-024-05432-0 (PMC12049378; doi:10.1007/s00345-024-05432-0)
Supplement: Supplementary file 2 — Supplementary file2 (DOCX 18 KB) [file 345_2024_5432_MOESM2_ESM.docx]

Supplementary Table 1. Other clinical characteristics after PSM.

| Characteristic | UUC | RPUC | p value |
| --- | --- | --- | --- |
|  | n = 199 | n = 199 |  |
| Gender, n (%) |  |  |  |
| Male | 143 (71.9) | 138 (69.3) | 0.58 |
| Female | 56 (28.1) | 61 (30.7) |  |
| Missing | 0 | 0 |  |
| Age (yr), median [IQR] | 70.0 [63.0-77.0] | 71.0 [64.0-77.0] | 0.59 |
| Smoking, n (%) |  |  |  |
| No | 69 (34.7) | 69 (34.7) | 0.84 |
| Ex-smoker | 68 (34.2) | 62 (31.2) |  |
| Current smoker | 45 (22.6) | 48 (24.1) |  |
| Missing | 17 (8.5) | 20 (10.1) |  |
| Race, n (%) |  |  |  |
| White | 139 (69.8) | 147 (73.9) | 0.42 |
| Asian | 45 (22.7) | 39 (19.6) |  |
| Other | 14 (7.0) | 9 (4.5)_ |  |
| Missing | 1 (0.5) | 4 (2.0) |  |
| Family history, n (%) |  |  |  |
| No | 159 (79.9) | 160 (80.4) | 0.66 |
| Yes | 8 (4.0) | 10 (5.0) |  |
| Missing | 32 (16.1) | 29 (14.6) |  |
| Contralateral UTUC, n (%) |  |  |  |
| No | 195 (98.0) | 197 (99.0) | 0.69 |
| Yes | 3 (1.5) | 2 (1.0) |  |
| Missing | 1 (0.5) | 0 |  |
| Solitary kidney, n (%) |  |  |  |
| No | 198 (99.5) | 199 (100.0) | —— |
| Yes | 0 | 0 |  |
| Missing | 1 (0.5) | 0 |  |
| Concomitant bladder cancer, n (%) |  |  |  |
| No | 128 (64.3) | 140 (70.4) | <0.01 |
| Yes | 35 (17.6) | 12 (6.0) |  |
| Missing | 36 (18.1) | 47 (23.6) |  |
| ASA score, n (%) |  |  |  |
| 0 | 21 (10.6) | 25 (12.6) | 0.71 |
| 1 | 101 (50.7) | 96 (48.2) |  |
| 2 | 71 (35.7) | 67 (33.7) |  |
| 3 | 4 (2.0) | 7 (3.5) |  |
| Missing | 2 (1.0) | 4 (2.0) |  |
| Adjuvant chemotherapy, n (%) |  |  |  |
| No | 185 (93.0) | 187 (94.0) | 0.54 |
| Yes | 14 (7.0) | 10 (5.0) |  |
| Missing | 0 | 2 (1.0) |  |
